# Persistent candida arthritis successfully treated with micafungin instillation and surgery. A case report

**DOI:** 10.1016/j.mmcr.2019.12.011

**Published:** 2019-12-17

**Authors:** Jeroen C. van Egmond, Nicole G.M. Hunfeld, Bart J.A. Rijnders, Jan A.N. Verhaar

**Affiliations:** a Department of Orthopaedics, Erasmus University Medical Center, Doctor Molewaterplein 40, 3015 GD, Rotterdam, the Netherlands; b Department of Pharmacy, Erasmus University Medical Center, Doctor Molewaterplein 40, 3015 GD, Rotterdam, the Netherlands; c Department of Intensive Care, Erasmus University Medical Center, Doctor Molewaterplein 40, 3015 GD, Rotterdam, the Netherlands; d Department of Infectious Diseases, Erasmus University Medical Center, Doctor Molewaterplein 40, 3015 GD, Rotterdam, the Netherlands

**Keywords:** Candida, Micafungin, Knee, Arthritis, Instillation

## Abstract

We report a rare case of C. krusei knee arthritis treated with instillation of micafungin and arthroscopy. A 49-year-old man hospitalized for treatment of Acute Myeloid Leukemia developed knee arthritis with C. krusei. He was treated with a combination of arthroscopic debridement, intravenous as well as intra-articular micafungin. Serum and intra-articular concentrations of micafungin were determined. After instillation of micafungin in the knee and arthroscopic debridement, the patient completely recovered.

## 1 Introduction

In the Netherlands, septic arthritis is a relatively rare disease with an incidence of 5,7 per 100.000 inhabitants per year [1], and in adults mostly affects the knee [2].

Urgent treatment of septic arthritis is necessary to prevent further irreversible joint destruction. In humans with intact immunity most septic arthritis are bacterial and caused by *S. aureus* [3]. In immunocompromised patients, Candida species can infrequently be the cause of an infectious arthritis as well [4]. As the number of patients with malignant hematological diseases that are receiving treatments with new immunotherapies and molecular targeted agents is increasing rapidly, more patients are at risk for invasive fungal infections [5]. The treatment of a septic arthritis caused by Candida is not well defined, although a successful outcome has been reported with oral or intravenous antifungal therapy with fluconazole, amphotericin-B and voriconazole [[6], [7], [8]].

However, few data are available about the pharmacokinetics of antifungal agents in synovial fluid and tissue and it is unclear if the achieved concentrations exceed the minimal inhibitory concentration (MIC) of Candida species. To attempt to achieve appropriate articular drug concentrations, instillation of amphotericin-B into the joint has been used as adjuvant therapy as well [9,10].

Recent guidelines on the treatment of invasive Candida infections recommend the use of fluconazole, an echinocandin or liposomal amphotericin-B for Candida arthritis [11]. In case of a fluconazole resistant Candida species like *C. krusei* an echinocandin or liposomal amphotericin-B are the only appropriate initial antifungal therapies available. To the best of our knowledge, intra-articular instillation of micafungin to treat a septic arthritis with Candida species has not been described. Here we report a case of knee arthritis with *C. krusei* treated with a combination of surgical debridement, intravenous as well as intra-articular micafungin.

## 2 Case

A previously healthy 49-year-old man who developed Acute Myeloid Leukemia (AML), was admitted to the Hematology department to initiate intensive remission-induction chemotherapy. During the subsequent chemotherapy-induced episode of febrile neutropenia his blood cultures became positive for *C. krusei*. Treatment with intravenous micafungin 100mg once daily was initiated. Prophylaxis with fluconazole was stopped and his central venous line was removed. *C. krusei* was also cultured from a weekly surveillance rectal swab culture and therefore, the most likely source of the candidemia was gastro-intestinal translocation. After his blood cultures became negative, intravenous micafungin was continued for another 2 weeks and he could be discharged.

A week later, the patient was readmitted for a second course of chemotherapy and amphotericin-B oral solution was given to prevent a new episode of translocation of *C. krusei*. During this hospital stay he hit his left knee on the side of the bed. His knee continued to be painful and as a knee effusion was noted three days after injury, the orthopedic surgeon was consulted. On physical examination a large effusion of his left knee was found. The joint was not visibly red, but was warm on palpation. His temperature was 38.4° Celsius. A plain knee radiograph of the knee was normal. An aspiration of the affected knee was performed and the joint fluid was sent for culture, this day was considered as day 0. Day 1 the joint aspiration grew *C. krusei* for which intravenous micafungin was restarted at a dose of 100mg twice daily. As the patient was deeply thrombopenic as well as neutropenic at that time, no arthroscopic drainage and lavage was performed but instead the knee was repeatedly drained by needle aspiration. On the day of the first needle aspiration of the knee and for the next 10 days, blood cultures were positive for *C. krusei* as well. Furthermore, repeated needle aspirations of the knee continued to grow *C. krusei*. An echocardiography at day 6 was normal. The ongoing candidemia and repeatedly positive synovial fluid cultures led to the decision at day 7 to instill micafungin in the knee at a dose of 1mg in 10ml saline. Furthermore, liposomal amphotericin-B was started at day 8 at 3mg/kg/day intravenously while intravenous micafungin was continued. After 4 days intra-articular injections were stopped. However, despite repopulation from chemotherapy induced neutropenia, his blood cultures as well as synovial fluid cultures continued to be positive. At day 13 a whole-body PET scan showed arthritis of the left knee, PET positive pulmonary infiltrates, and some pleural fluid. Since the patients was no longer cytopenic the decision was made at day 17 to perform an arthroscopic debridement and lavage of the knee with an extensive arthroscopic synovectomy. Furthermore, the intra-articular administration of micafungin was reinitiated and continued for 7 days with at a dose of 3 mg per instillation (as a solution of 1mg in 10ml sodium chloride 0.9%). From this point onwards his blood cultures remained negative and he improved clinically. Further follow-up of his left knee revealed good recovery with less pain and good function six months postoperative.

*C. krusei* has been identified with the use of CHROMagar™ with subsequent identification by Maldi-TOF, with database MALDI BDAL Database version 8.0. Micafungin concentrations were measured (laboratory of pharmacy, University Hospital Nijmegen, the Netherlands) in serum and joint fluid [12]. The concentrations in serum 12 hours after the administration of 100mg intravenously at day 13 and 16 of therapy were 4.5 and 1.5mg/L respectively and concentration in synovial fluid 24 hours after installation of 3mg of micafungin in the knee was 11mg/L at day 19, as well presented in Table 1. Reference concentrations of micafungin in serum derived from 2 studies in critically ill patients trough level 1.4–3.1 mg/L were described [12,13]. In healthy subjects, micafungin has as a volume of distribution of 0,2L/kg with a half-life of 15 hours. Administration of 100mg once daily results in trough serum concentration levels of 2 mg-L [14]. In hematology patients reference concentrations after intravenous micafungin are available as well [15].

**Table 1:** Administration of micafungin and micafungin concentrations over time.

Day of administration micafungin	Route of administration	Dose	Micafungin concentration (mg/L)
day 1 – day 15	IV	100mg BID	
day 7 – day 10	IA	3–10 – 20–20 mg	
day 14			4.5 (trough serum)
day 16 – day 32	IV	200mg OD	
day 17^b^			1.5 (trough serum)
day 17 – day 24	IA	3mg OD	
day 19			11 (joint)^a^

Abbreviations: IA: intra-articular, IV: intravenous, BID: two times daily, OD: one time daily.

a Aspiration of joint fluid 24 hours after administration of micafungin (trough concentration).

b Day of surgery.

## 3 Discussion

We present a rare case of knee arthritis caused by *C. krusei* during chemotherapy induced neutropenia in a patient with AML. He was successfully treated with a combination of surgical debridement, intravenous as well as intra-articular micafungin.

Micafungin is an echinocandin, registered for the treatment of Candida infections. Despite intravenous therapy with micafungin and repeated needle aspiration of the knee, blood as well as synovial fluid cultures continued to be positive in our patient. Eventually, the combination of surgical debridement and the intra-articular instillation of micafungin led to clinical resolution of the infection.

Patients undergoing intensive chemotherapy for hematological malignancies often receive prophylaxis against invasive Candida infections with fluconazole. However, breakthrough candidemia with fluconazole resistant candida species, like *C. krusei* are occasionally observed [16]. In these patients, Candida arthritis is typically the consequence of hematogenic dissemination [6].

Lu et al. presented a case report of knee arthritis with *C. krusei* and reviewed the available literature on 4 other cases [17]. Given the 30-year time frame of these 5 cases, it is not surprising that the antifungal therapy given to these patients was diverse and only 2 received an echinocandin. Our patient was treated with surgical debridement in combination with intravenous and intra-articular micafungin for a total of 12 days.

Synovial fluid is a transudate of plasma, so dissolution of micafungin in synovial fluid will be likely. Moreover, diffusion from plasma to the synovial fluid will occur. Unfortunately, data about the extent of diffusion from plasma to the synovial fluid are not available. The possible side effects as well as the possible toxic effects on cartilage of micafungin when administered directly into a joint are unknown. However, we decided to administer micafungin through direct intra-articular instillation as, despite prolonged high-dose intravenous micafungin therapy, blood as well as synovial cultures remained positive in our patient. Reconstitution of micafungin in NaCl 0.9% results in a pH of 5–7 [18]. Further dissolution in NaCl 0.9% will results in a pH of about 7, with isotonic properties. A dose of 3mg per instillation was given from day 17–24 as a 1mg/10ml NaCl 0.9% solution. This dose was anticipated to result in local concentrations well above the MIC of the *C. krusei* (0.125 μg/ml) for a sufficiently long time and this was confirmed by the measured micafungin concentration in synovial fluid of 11mg/l 24 hours after instillation, a concentration several times higher than what was measured in blood in the same patient (Table 1).

Toxicity of the joint is one of the main concerns after local injection. Despite the high local concentration of 11mg/l, no local toxicity was observed as the patient recovered with minor complaints of his knee.

Besides anti-bacterial or anti-fungal medication, load reduction is key in patients with septic arthritis and can be performed by arthroscopy or arthrotomy. Arthroscopic treatment is preferable to arthrotomy in terms of pain, rehabilitation and range of motion [19]. Surgical irrigation and debridement is not always needed in mild disease burden, although the literature is lacking on this and consists of case reports [6].

In conclusion, instillation of micafungin in the knee of a patient with therapy resistant *C. krusei* arthritis led to therapeutic synovial concentrations and was used successfully in combination with surgical debridement.
